# Prevalence of HPV infection in the general population of young and adult males in Italy

**DOI:** 10.1111/andr.13817

**Published:** 2024-12-03

**Authors:** Giuseppe Grande, Andrea Graziani, Luca De Toni, Federica Finocchi, Adriano Presciutti, Sara Corrò, Alberto Ferlin, Andrea Garolla

**Affiliations:** ^1^ Unit of Andrology and Reproductive Medicine, Department of Systems Medicine University Hospital of Padova Padova Italy; ^2^ Department of Medicine University of Padova Padova Italy

**Keywords:** HPV, infection, prevalence, vaccination, young

## Abstract

**Background:**

The most prevalent sexually transmitted disease in the world has the human papillomavirus (HPV) as its etiological agent.

**Objectives:**

To evaluate the prevalence of previous and actual HPV infection and the clinical manifestations in unselected males.

**Materials and methods:**

A total of 718 males participating to a surveillance program were asked to complete a study visit at our unit, including semen collection, balanopreputial sulcus swab, and blood collection for total anti‐HPV immunoglobulin G (IgG). When HPV‐DNA was detected, we performed HPV fluorescence in situ hybridization, oral and anal swab, and penoscopy. Because previous studies demonstrated a very high risk for HPV infection in subjects with history of HPV‐induced lesions, with a partner with diagnosed HPV infection or reporting couple infertility or sexual promiscuity and an increase of the risk in males having sex with males, in subjects with unprotected sexual intercourses or in heavy smokers, patients were therefore stratified according to the presence of these known risk factors (RFs).

**Results:**

Actual HPV infection was detected in 401/718 subjects (55.85%). Oral HPV‐DNA was reported in 80 subjects and anal HPV infection in 52 subjects. Anti‐HPV IgG antibodies have been detected in 288 subjects. The overall prevalence of HPV exposition, considering actual and/or previous infection was 77.99%. Among infected men, high‐risk HPV genotypes were detected in 66.08%.

A total of 514 subjects were considered as the RF population, while 150 were classified in the non‐RF population. There was a significantly higher prevalence of condylomatosis (odds ratio [OR] 4.07) and of seminal infection (OR 6.22) in the RF group.

**Discussion and conclusion:**

These data represent an alert for the healthcare system to perform informative and screening campaigns for HPV infection in males and to promote HPV vaccination both in young people and for adult males with RF for HPV infections.

## 1 INTRODUCTION

Infection with the human papillomavirus (HPV) is widespread in both genders and occurs in all racial, social, and geographic contexts. There are an estimated 6.2 million new instances of HPV infection each year, making it the most common sexual transmitted infections (STI) globally.^1^ HPV comprises a group of small non‐enveloped epitheliotropic viruses with a double‐stranded circular DNA genome made up of 8000 bp. With a diameter of 55 nm, the virion is icosahedral in shape and is made up of 52 capsomeres, each of which has five molecules of the major capsid protein L1 and less molecules of the minor capsid protein L2.^2^ More than 200 genotypes of HPV are known to be specific to certain epithelial tissues, including the mucosa and anogenital skin.^3^

HPV can be classified into two types based on their carcinogenic potential: low‐risk (LR‐HPV) and high‐risk (HR‐HPV). Plantar, flat, or cutaneous warts are thought to be caused by LR‐HPV subtypes.^4^ Additionally, condyloma acuminata and oral and anogenital warts can be caused by various mucosal LR‐HPV strains.^5^ Because of the close connection to warts, HPV was once referred to as the “human warts virus.”^6^ Consequently, benign papillomas or subclinical infections are typically caused by LR‐HPV.^7^

HR‐HPV subtypes include 16, 18, 31, 33, 35, 39, 45, 51, 52, 56, 58, 59, 68, 73, and 82.^8^ Types 16 and 18 are the two most common causes of HPV‐related malignancies, which include cancers of the cervical, anal, penile, and oropharyngeal regions.^9^

In addition, types 26, 53, 66, and 69 have been reported by Muñoz et al.^8^ as probably carcinogenic; furthermore, the International Agency for Research on Cancer (IARC) includes genotypes 30, 34, 69, 85, and 97 among the potentially carcinogenic.^10^ We can therefore consider all these genotypes as at intermediate risk.

The IARC has stated that there is compelling evidence that HPV is a factor in the development of cancers in the cervix uteri, penis, vulva, vagina, anus, and oropharynx.^11^ Because HPV causes around 30% of all malignancies linked to infectious agents, the virus plays a significant role in the genesis of cancer. About 5% of cancer occurrences worldwide are caused by HR‐HPV subtypes, with an estimated 60,000 men and 570,000 women contracting the virus annually.^12^

Although previously, research on HPV‐related illnesses has primarily focused on females, there has been a recent surge in interest in HPV infection in males.^13^ In this sense, men are more likely than women to get HPV oral infections,^14^ and it is important to keep in mind that men are also more likely than women to get oropharyngeal cancer and it is to remember that oropharyngeal cancer mostly occurs in male patients too.^15^ The most frequent location of head and neck cancer linked to HPV is the oropharynx.^13^ Specifically, lesions appear at lymphoepithelial locations such as the base of the tongue and the palatine tonsil.^16^ It is thought that the lymphoid tissue at the base of the tongue, the crypts, and the uneven tonsil surface, all contribute to the persistence of HPV infection.^17^ This factor seems to be especially important for HPV persistence, as it raises the possibility of developing oropharyngeal squamous cell cancer (OSCC).^13^, ^17^

Moreover, it has now been conclusively shown that HPV virions can also be found inside the male reproductive tract, in addition to the well‐known exterior genital sites. Semen has been found to contain HPV‐DNA in both exfoliated cells and spermatozoa.^18^ The precise location and mechanism of sperm‐HPV binding have been elucidated by the use of immunofluorescence methods.^19^ In details, the HPV‐L1 capsid protein can connect to the glycosaminoglycan Syndecan‐I on the surface of spermatozoa and position itself in the head's equatorial region. In these conditions, HPV semen infection is associated with asthenozoospermia, male infertility, and recurrent miscarriages, thus representing an impairing factor both for natural and for assisted fertility outcome, through different mechanisms, including reduced sperm motility, development of anti‐sperm antibodies, and increase in sperm DNA fragmentation.^18^, ^20^, ^21^, ^22^ Recently, the European Society of Human Reproduction and Embryology (ESHRE) included HPV semen infection among viral infections which might impair the success of assisted reproduction.^23^ In terms of the epidemiology of HPV infection, there is a clear distinction between the prevalence of the virus in males and females: in the former, the prevalence rises during the first few years following the onset of sexual activity and then declines, whereas in the latter, the prevalence rises continuously throughout life.^24^

Starting from these premises, preventative HPV vaccinations are a vital weapon in the fight against infections, warts, and cancers brought on by the most common varieties of these viruses. Adolescent girls between the ages of 9 and 14 should have an HPV vaccination, according to vaccination programs implemented in several countries. In certain cases, boys should also receive this vaccination. Adult immunization is also taken into consideration by the programs, especially for those with various immunodeficiencies. Since 2015, HPV vaccination is proposed for free in Italy for young males and females at the age of 11 years.

HPV vaccination has been moreover proposed as an adjuvant treatment in infertile patients with HPV infection, because it enhances HPV healing in semen cells and increases rate of natural pregnancies and live births.^25^

The current study's aim was to evaluate the prevalence of previous and actual HPV infection and the related clinical manifestations in a population of unselected males and their relations with risk factors (RFs) for HPV.

## 2 MATERIALS AND METHODS

### 2.1 Participants

From January 1, 2022 until June 30, 2023, we conducted an andrological surveillance program in young and adult males, to inform, assess and reduce andrological RFs for each specific range of age, including HPV infection. The campaigned achieved sampling 1600 males. We proposed to the participants to be enrolled in a screening protocol about HPV infection and 718 males, aged 16–45‐year old, agreed to be enrolled for this study, including 70 young subjects aged 16–20 years and 648 adult subjects aged 21–45 years.

Having a malignancy was the only exclusion criterion. Eleven subjects had completed HPV vaccination. Participants were asked to complete a study visit at our Unit, including semen collection, balanopreputial sulcus swab, and blood collection to measure serum total anti‐HPV immunoglobulin G (IgG) levels and determine titers. All participants filled a questionnaire about demographic characteristics, medical history, lifestyle, and sexual habits, evaluating in details previous history of penile lesions, history of infection in the partner, history of infertility, smoking, sexual activity with men, casual sexual activity without use of condom, and history of sexual promiscuity (more than three sexual partners in the last month).

Balanopreputial sulcus swab and semen collection by masturbation were analyzed for HPV detection and genotyping. A peripheral blood sample for anti‐HPV antibodies assay was collected in all subjects.

When HPV‐DNA was detected in semen or balanopreputial sulcus swab, we performed HPV fluorescence in situ hybridization (FISH) for HPV, oral and anal swab, and penoscopy.

Written informed permission was acquired by each subject. The Institutional Review Board gave their approval to the study protocol.

### 2.2 HPV detection and genotyping

HPV‐DNA detection and genotyping were performed on balanopreputial sulcus swab (Copan Italia S.p.A.) and in semen samples by INNO‐LiPA Genotyping Extra assay, permitting the identification of the HPV types: 6, 11, 16, 18, 26, 31, 33, 35, 39, 40, 42, 43, 44, 45, 51, 52, 53, 54, 56, 58, 59, 66, 68, 69, 71, 70, 73, 74, and 82.

HPV subtypes 16, 18, 31, 33, 35, 39, 45, 51, 52, 56, 58, 59, 68, 73, and 82 have been considered as HR‐HPV. HPV genotypes 26, 53, 66, and 69 have been considered as at intermediate risk. The remaining genotypes have been considered as LR‐HPV.

### 2.3 Anti‐HPV antibodies

Using a commercial kit provided by DRG Diagnostic GmbH, the detection and titer of serum total anti‐HPV IgG were evaluated on serum samples from peripheral blood taken from all patients, as previously described.^24^ The analysis was conducted using an enzyme‐linked immunosorbent assay (ELISA).

### 2.4 HPV fluorescence in situ hybridization

Percoll gradient centrifugation was used to separate the sperm cells from the native semen samples, and sterile phosphate‐buffered saline (PBS) was used for the three rounds of washing. FISH for HPV in spermatozoa was carried out as previously mentioned.^20^ In summary, samples were fixed for at least an hour at −20°C in a methanol–acetic acid solution (3:1, vol/vol). Following dehydration and permeabilization, samples were covered with 20 mL of hybridization solution (Pan Path), which contained an HPV‐DNA probe labeled with biotin (a mixture of whole genomes that contain the conserved HPV region). After that, a glass cover slip was placed over each slide, and the HPV‐DNA probe and denaturation of the cellular target DNA were carried out. After that, slides were once more cleaned three times in PBS before being incubated with the differentiation reagent (Pan Path). By incubating 1:200 streptavidin Texas red (Vector Laboratories) for 40 min at room temperature, the biotin‐labeled HPV probe could be identified. The slides were cleaned twice in PBS/0.01% Triton (Sigma‐Aldrich) and then twice in PBS after detection. Nuclei were counterstained with a solution containing 6‐diamino‐2‐phenylindole (DAPI, 5 mg/mL), slides were mounted with antifade buffer (BioBlue; BioView) and 24–24‐mm cover slips. Samples were finally analyzed using a fluorescence microscope (Eclipse E600, Nikon) equipped with a triple band‐pass filter set (fluorescein isothiocyanate conjugate [FITC], tetramethyl rhodamine isothiocyanate, and DAPI). At least 200 spermatozoa were examined on each slide. Three researchers evaluated the results of nuclear hybridization. Tests of the procedure were conducted on positive control slides that included CaSki cells, a human cervical cancer cell line with transcriptionally active and stably integrated HPV genomes that acted as a control for the particular probe. The viral probe was left out of the negative control, which was handled in the same way.

### 2.5 Penoscopy

After applying 3% acetic acid to the selected group of patients, a skilled research physician used a colposcope (magnification factor: ×0.6–1.6; Zeiss) to perform a penoscopy. Digital photos were used to document penile lesions, which were classed as flat penile lesions or condylomatosis acuminata.

### 2.6 Stratification for the presence of risk factors

Previous studies demonstrated a very high risk for HPV infection in subjects with history of HPV‐induced lesions (odds ratio [OR] 17.9),^18^ with a partner with diagnosed HPV infection (OR 28.4)^18^ or reporting couple infertility (OR 4.6)^18^ or sexual promiscuity (OR 3.0).^26^ Furthermore, an increase of the risk for HPV infection has been previously described in the population of males having sex with males (MSM; OR 1.6),^27^ in subjects with casual sexual intercourses not always using condom (OR 1.5)^26^ or in heavy smokers (>15 cigarettes per day; OR 1.2).^28^

According to these previous data, we aimed to verify how the presence of previously reported RFs in the history of patients may modify the risk of HPV infections and the prevalence of the clinical manifestations. We therefore divided the study group based on the presence of known RFs for HPV infection, as reported in Table 1. We included in the group with RFs subjects with almost one factor included in the group A, highly associated with HPV infection (history of genital warts, partner with HPV infection, infertility, and sexual promiscuity) or with almost two factors included in the group B (MSM population, unprotected casual sexual intercourses, or smoking).

**TABLE 1:** Classification for risk factors according to previous evidences.

**Group A**
History of genital warts
Partner with HPV infection
Infertility
Sexual promiscuity
**Group B**
MSM population
Unprotected intercourses
Heavy smoking

Abbreviations: HPV, human papillomavirus; MSM, males having sex with males.

### 2.7 Statistical analysis

Patients were stratified in groups according to the age (<20 years vs. ≥20 years) and the presence of known RFs for HPV infection. Chi‐square test was used to compare the prevalence of HPV infection or exposition among the studied groups. Statistical significance was assumed if *p* values were ≤0.05.

## 3 RESULTS

Mean ± SD age of the studied population was 34.28 ± 9.69 years.

HPV has been detected in semen in 400/718 (55.71%) subjects and in balanopreputial sulcus in 401 subjects (55.85%). In particular, the concordance between semen and balanopreputial sulcus swab was >99%, as concordance was missing only in 1/401 patients (positivity at balanopreputial sulcus and negativity in the semen). Oral HPV‐DNA was reported in 80/401 (20%) subjects and anal HPV infection in 52/401 (13%) subjects with HPV infection in semen and balanopreputial sulcus swab.

Anti‐HPV IgG antibodies have been detected in 288 subjects (40.1%), including 129 subjects with HPV‐DNA detection (129/401, 32.2%) and 159 subjects without current infection (159/317, 50.2%).

Penile lesions at penoscopy were observed in 14.5% (104/718) of the total population. In all patients with penile lesions HPV‐DNA was detected in semen and/or balanopreputial sulcus swab; in 17 patients with penile lesions we reported the presence of anti‐HPV antibodies.

**FIGURE 1:**
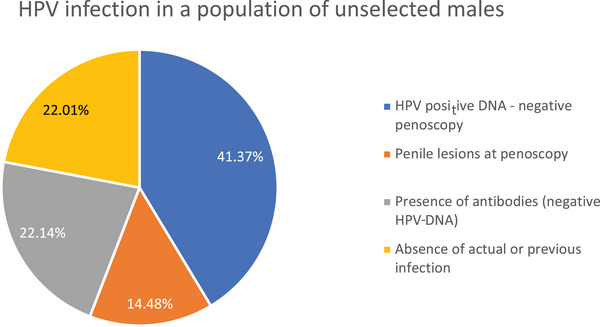
Prevalence of human papillomavirus (HPV) current or previous infection in the studied population.

The overall prevalence of HPV infection, considering actual (HPV‐DNA and/or penile lesions) and/or previous (presence of anti‐HPV IgG antibodies) infection, was therefore 77.99% (560/718), as reported in Figure 1.

**FIGURE 2:**
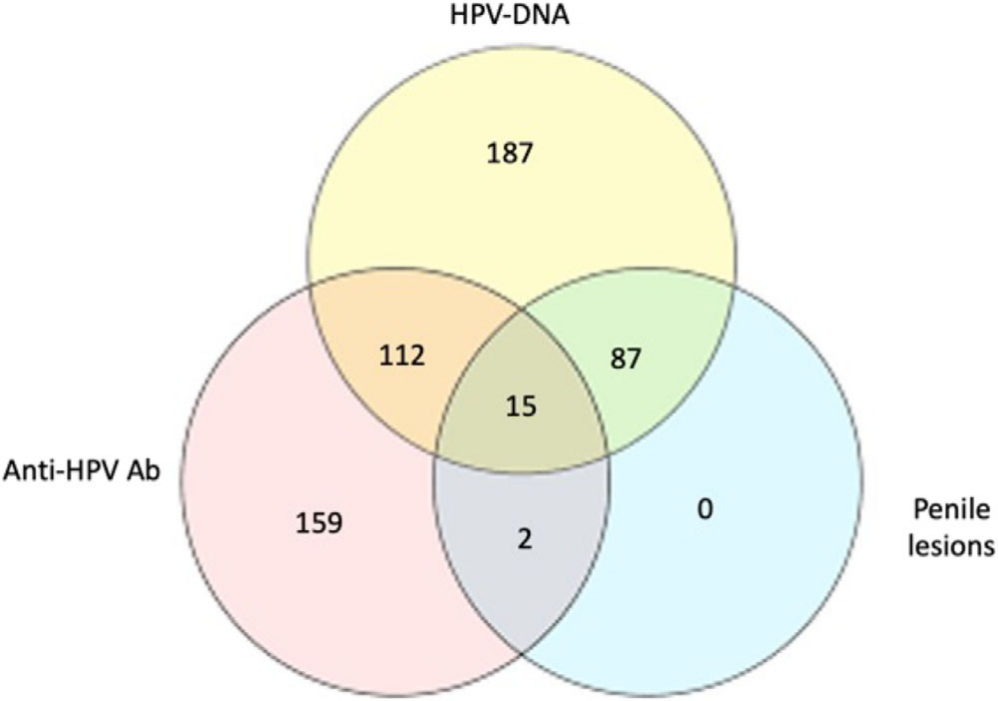
Relative prevalence of human papillomavirus (HPV)‐DNA, anti‐HPV antibodies (Ab) and penile lesions in the studied populations.

Figure 2 reports the distribution of patients with penile lesions, HPV‐DNA, and anti‐HPV antibodies.

Among infected men, HR‐HPV genotypes were detected in 265/401 subjects (66.08%). Forty‐four subjects (10.97%) had genotypes associated with intermediate risk, while 22.94% had LR HPV. Table 2 reports the relative prevalence of each genotype included in the HPV vaccine. In details, 81/401 infected subjects (20.20%) had a genotype included in the nonavalent HPV vaccine.

**TABLE 2:** Frequency of the different human papillomavirus (HPV) genotypes included in nonavalent HPV vaccine, identified in semen and/or balanic swab.

HPV genotype	Number of patients positive for HPV‐DNA
6	11
11	8
16	19
18	8
31	10
33	3
45	7
52	12
58	3

After classification for known RFs for HPV infection, 514 subjects were considered as the RF population, while 150 were classified as part of the non‐RF population.

Demographic data and prevalence of penile lesions, HPV‐DNA detection and presence of anti‐HPV antibodies in the two groups are reported in Table 3.

**TABLE 3:** Age and prevalence of human papillomavirus (HPV) infection in risk factor (RF) and non‐RF groups.

	RF group (*n* = 514)	Non‐RF group (*n* = 150)
Age (mean ± SD) (years)	34.71 ± 7.90	34 ± 12.81
Current infection (penile lesions + HPV positive DNA)	325 (63%)^*^	76 (51%)
Penile lesions at penoscopy; *n* (%)	97 (19%)^*^	7 (5%)
Presence of HPV antibodies (negative HPV‐DNA)	158 (31%)	41 (27%)
Absence of infection	31 (6%)^*^	33 (22%)

* *p* < 0.01.

Among subjects with current infection (RF *n* = 325; non‐RF *n* = 76), a significative higher prevalence of condylomatosis has been reported, with an OR for the RF group of 4.07 (95% confidence interval [CI] 1.8043–9.1903, *p* < 0.005). Infected subjects in RF had more frequently seminal infection (228/325 in RF; 19/76 in non‐RF group; *p* < 0.01). The presence of RF was associated with an OR of seminal HPV‐DNA detection of 6.22 (95% CI 3.5275–10.9612, *p* < 0.0005).

**FIGURE 3:**
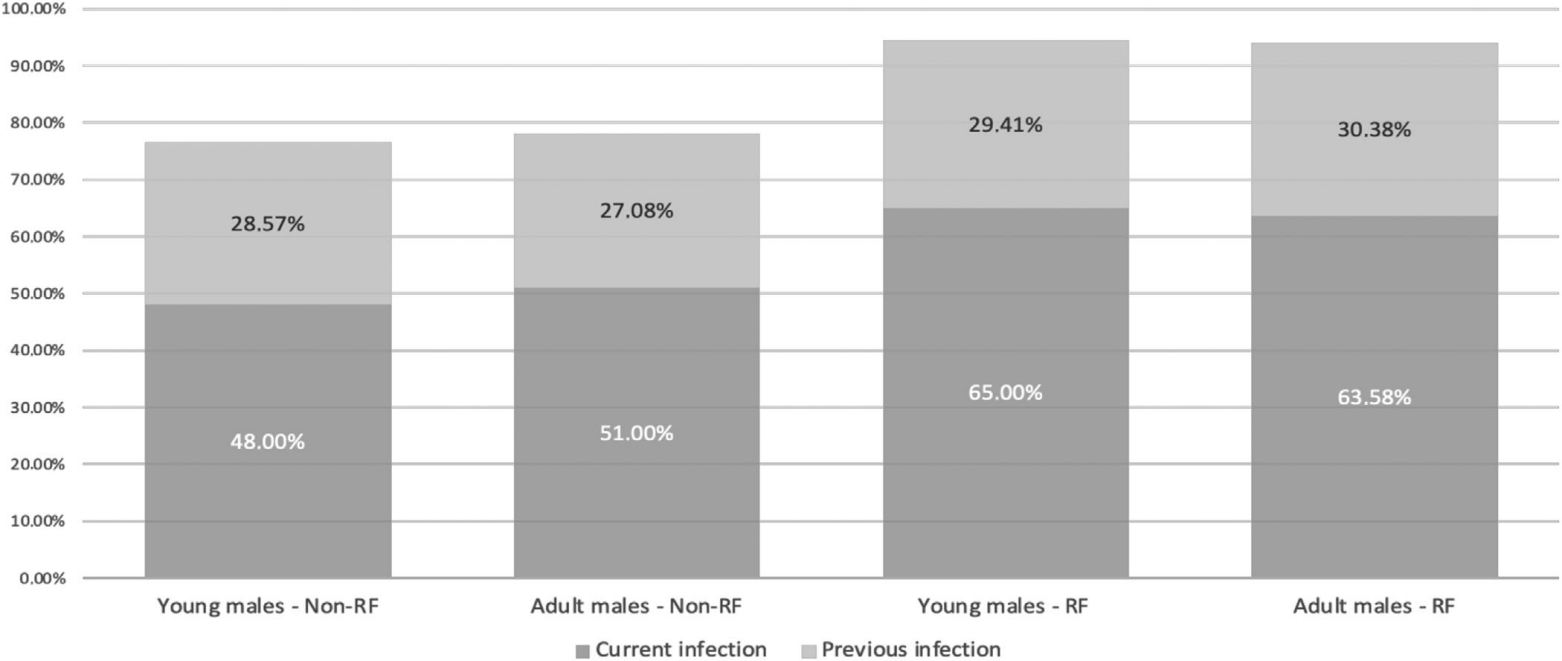
Prevalence of human papillomavirus (HPV) infection in the cohort of young males (16–20‐year old) and in the group of adult males (20–45‐year old) with and without risk factors (RFs) for HPV infection.

We finally divided the study population according to the age. In details, we analyzed data from 70 young males, 32 in the FR group and 38 in the non‐FR group. Only 11 subjects had completed HPV vaccination (15.7%): seven subjects in the FR group (21.8%) and four in the non‐FR group. All vaccinated subjects developed anti‐HPV antibodies and HPV‐DNA was not identified in any of these subjects. When considering unvaccinated subjects, as reported in Figure 3, no significant differences have been reported for the prevalence of HPV current and previous infection in the group of young and in the group of adult males after stratification for the presence of RFs.

## 4 DISCUSSION

Many viruses can infect the male reproductive tract, with potential adverse consequences to male reproductive health, including infertility and cancer.^29^ Among them, recent insights revealed the role of male HPV infection in cancerogenesis and human reproduction.^30^

Epidemiologic information on genital HPV infection, especially in the male population, is sparse because most infections are subclinical. Previous epidemiologic studies performed in US in women demonstrated that 1% of women have genital warts. In another study about 33% of women had HPV infection detectable by PAP smears,^31^ while the prevalence of positive HR‐HPV types 16 and 18 DNA test in healthy women with negative Pap smears is estimated in another 10%.^32^ In conclusion, the estimated prevalence of active HPV infection in women is about 44%. Furthermore, the seroprevalence of HPV 16 and 18 was reported in 24% of HPV‐DNA negative women,^33^ so that the final exposition to HPV infection in women (including actual and previous infection) may be estimated in 68% of the general population.

Few studies have been performed to evaluate the overall prevalence of HPV infection and exposition in male. There is a wide range of reported prevalence of genital HPV‐DNA in men (≥20% in most studies), ranging from 1.3% to 72.9%. The most likely causes of these differences include variations in the populations examined, in the sampling sites, in the detection strategies employed, and in the sampling methodologies. The estimate of HPV prevalence has increased recently due to the use of more sensitive sampling methods and to sample collection from a larger number of anatomic locations. More than 60% of men tested positive for at least one HPV genotype, according to an international survey.^34^ In 2014, Lorenzon et al. reported a prevalence of HPV infection in the Italian healthy male population of 40.5%.^35^

We confirmed the alarming data about the high prevalence of actual HPV infection in 55.85%, of the male population, when HPV‐DNA is searched in semen and on balanopreputial sulcus swab. It means that one out two healthy men in the general population has an active HPV infection.

The prevalence of penile lesions has been reported in about 15% of the population, using penoscopy. It is a widely used diagnostic procedure in clinical practice for screening HPV lesions. It is relevant, but not conclusive diagnostic tool.^36^ False positivity from the acetic acid test is mainly due to inflammatory or confounding conditions. The predictive power of penoscopy, however, is very high especially when correlated with histopathological findings or, as done in the present study, with HPV‐DNA test.^36^, ^37^

After considering the seroprevalence for HPV, we can affirm that 78% of an unselected male population had entered in contact with HPV.

HR‐HPV genotypes have been reported in about 66% of the patients with active infection, underlining that these subjects (and their partners) are at risk of carcinogenesis. Important oncogenes for HPV carcinogenesis, HPV E6 and E7 are in fact frequently found in conjunction with HR‐HPV types.^38^, ^39^, ^40^

Within the basal epithelium, HPV begins the cellular sabotage phase by reprogramming the host cell machinery to continue replication; the next step is characterized by the release of HPV from terminally differentiated cells that detach from the epithelial surface.^41^ According to pathophysiology, the point at which HPV integrates into the host genome is when oncoproteins E6 and E7 begin to activate. These proteins are linked to the degradation of p53 and pRb, respectively, which in turn causes an increase in the rate of proliferation and tumor growth‐promoting transcription factors.^42^

Starting from these physiopathological premises about the role of HR‐HPV genotypes in carcinogenesis and considering the high prevalence of HR‐HPV infection reported by these data, the urgency of creating and executing a comprehensive, coordinated, and gender‐neutral international strategy to eradicate HPV‐related diseases in Europe through widespread vaccination and youth education campaigns is thus called into question.

Furthermore, when stratifying patients according to their belonging to the group with RFs for HPV infection, the prevalence of HPV infection arises to 63%. Patients with RFs have more frequently condylomatosis and particularly seminal infection, which has been previously demonstrated to be associated with infertility and frequent miscarriages.

Finally, the high prevalence of HPV infection is observed from a young age (16–20‐year old) and no differences in the prevalence of HPV actual or previous infection has been observed in the group of young people when compared with adults, with or without RFs, thus confirming the importance of early vaccination of young people for HPV infection.

## 5 CONCLUSIONS

Despite the limits of the study, performed on unselected male subjects involved in an andrological surveillance program based on a voluntary basis, thus characterized by the absence of specific information, such as data about foreskin status, seminal status, and follow‐up at penile lesion biopsy, all these data, taken together, represent an alert for the healthcare system to perform screening campaigns for HPV infection in males, to promote informative campaigns about HPV‐associated diseases, HPV‐associated RFs, and correct lifestyles, to promote HPV vaccination in young people, and to internationally extend HPV vaccination including adult males with RF for HPV infections.

## AUTHOR CONTRIBUTIONS

Giuseppe Grande and Andrea Garolla designed the study; Luca De Toni, Federica Finocchi, Adriano Presciutti, and Sara Corrò have helped with analysis and data acquisition; Giuseppe Grande, Andrea Graziani, and Andrea Garolla analyzed the data; Giuseppe Grande and Andrea Graziani drafted the paper; Alberto Ferlin revised critically the manuscript. All the authors approved the submitted and final versions.

## CONFLICT OF INTEREST STATEMENT

The authors declare no conflicts of interest.

## Data Availability

The data that support the findings of this study are available from the corresponding author upon reasonable request.
